# Why Tolerate Conscience?

**DOI:** 10.1007/s11572-014-9325-2

**Published:** 2014-11-08

**Authors:** François Boucher, Cécile Laborde

**Affiliations:** London, United Kingdom

**Keywords:** Legal exemptions, Freedom of religion, Freedom of conscience, Toleration, Brian, Leiter

## Abstract

In Why Tolerate Religion?, Brian Leiter argues against the special legal status of religion, claiming that religion should not be the only ground for exemptions to the law and that this form of protection should be, in principle, available for the claims of secular conscience as well. However, in the last chapter of his book, he objects to a universal regime of exemptions for both religious and secular claims of conscience, highlighting the practical and moral flaws associated with it. We believe that Leiter identifies a genuine and important contemporary legal and philosophical problem. We find much to admire in his reasoning. However, we raise questions about two claims that are crucial for his argument. The first claim is that it is not religion as such, but conscience that deserves toleration and respect. The second claim is that respect for religion and conscience demands ‘principled toleration’ but does not entail stronger policies of legal exemptions. Against the first claim, we argue that Leiter does not successfully distinguish religious belief from secular conscience and morality; and he does not explain why secular conscience (which shares many of religious conscience’s epistemic features) deserves respect. Against the second claim, we argue that the most promising theories of legal exemptions are not classical theories of liberal toleration.

In his stimulating book, *Why Tolerate Religion?*,^1^ Brian Leiter focuses on the problem raised by the singling out of religion for special constitutional protection. In several Western legal systems, religious beliefs and practices are treated differently from secular, non-religious commitments and identities, be they cultural, ethical, artistic, scientific, linguistic, and so on. Western legal systems in practice single out religion for special treatment; they both grant religion special protection which is not extended to non-religious ethical fundamental commitments and impose special disabilities on religions which do not burden non-religious organizations and non-religious worldviews.^2^

Religion is burdened by special legal disabilities in countries with constitutional non-establishment clauses. For instance, in the United States, the government may not support and subsidize religious activities—yet no such ban applies to culture or the arts. Moreover, religious practices enjoy special legal protection in the form of legal exemptions from some general laws. By contrast, such protection is rarely available for non-religious practices. For instance, in Canada, the Royal Canadian Mounted Police allows members of the Sikh community to depart from its traditional uniform in order to wear the turban.^3^ The Supreme Court decided, in the *Multani* decision, that a Sikh schoolboy could wear the *kirpan*, a ritual dagger, into the classroom despite the school Board’s regulations forbidding students to bring knives in the classroom. However, no exemption from uniform rules on appropriate headgear is available for, say, dedicated baseball fans wishing to wear baseball caps at work or in the classroom.^4^

In his book, Leiter focuses on the special legal protection of religion. The puzzle that such special treatment of religion gives rise to is that:

no one has been able to articulate a credible principled argument for tolerating religion *qua* religion—that is, an argument that would explain why, as a matter of moral principle, we ought to accord special legal and moral treatment to religious practices. There are, to be sure, principled arguments for why the state ought to tolerate a plethora of private choices and conscientious commitments, as well as related practices of its citizenry, but none of these single out religion for anything like the special treatment it is accorded in existing Western legal system. So why tolerate religion? (p. 7).

Principled arguments for religious toleration are in fact arguments for tolerating those personal commitments that are grounded in the individual’s conscience. These are commitments about what one must do no matter what, not out of self-interest but ‘because it is a kind of moral imperative central to one’s integrity as a person, to the meaning of one’s life’ (p. 95, see also p. 34).

Many legal theorists and philosophers have recently drawn attention to the puzzling fact that law gives special treatment to religion, and asked whether other conscientious commitments should benefit from similar protection as religious conscience by virtue of their similarity with it. They have asked what counts as religion, whether religion should be viewed as special by the law, and whether non-religious commitments analogous to religious claims should benefit from equal protection.^5^

In *Why Tolerate Religion?*, Leiter offers an incisive and spirited contribution to this debate. He argues against the special legal status of religion, claiming that religion should not be the only ground for exemptions and that this form of protection should be, in principle, available for the claims of secular conscience as well. However, in the last chapter, he objects to a universal regime of exemptions for both religious and secular claims of conscience, highlighting the practical and moral flaws associated with it. In general, he defends the view that, although exemptions should be available for secular and religious conscientious claims alike, there should be no ‘burden-shifting exemptions’ (p. 99).^6^

We believe that Leiter identifies a genuine and important contemporary legal and philosophical problem. We find much to admire in his reasoning. However, we raise questions about two claims that are crucial for his argument. The first claim is that it is not religion as such, but *conscience* that deserves toleration and respect. Leiter argues that the defining features of religion are that it contains beliefs that make categorical demands upon believers and that are insulated from reasons and evidence. Yet, it is difficult to see why the latter feature should deserve any particular respect in itself; and so *whatever is respectable about religion is not specific to religion but is rather a manifestation of conscience, which can also take a secular form (claim 1)*. The second claim is that respect for religion and conscience demands ‘principled toleration’ but does not entail stronger policies of legal exemptions. This is because *the only way to justify legal exemptions is* via *classical theories of liberal toleration (claim 2)*.

In what follows, we dispute both claim 1 (the relevant features of both religion and conscience) and claim 2 (the appropriate justification for legal exemptions). Against claim 1, we argue that Leiter does not successfully distinguish religious belief from secular conscience and morality; and he does not explain why secular conscience (which shares many of religious conscience’s epistemic features) deserves respect. Against claim 2, we argue that the most promising theories of legal exemptions are not classical theories of liberal toleration. The next two sections analyze both claims—the claim about religion and conscience, and the claim about toleration—in turn.

## Religion and Conscience

Before examining whether religion should be tolerated *qua* religion, Leiter attempts to explain what the expressions ‘religion as such’ and ‘religion *qua* religion’ could mean. Leiter argues that what makes religion unique and special is that in all religions there are at least some central beliefs that 1) issue in categorical demands and 2) are insulated from evidence and reasons (p. 33–34). Categoricity and insulation from reasons and evidence are thus the two central distinctive features of the religious conscience. The latter epistemic feature of religion, according to Leiter, specially singles out religion *qua* religion and distinguishes it from other types of beliefs placing categorical demands upon individuals, such as those contained in political ideologies and secular moral codes.

Our goal in this section is to show that Leiter fails to identify the features which demarcate religious conscientious commitments from non-religious ones. We demonstrate that Leiter fails to establish insulation from reasons and evidence as the demarcating feature of religion. This is because he draws on incompatible interpretations of ‘insulation from reasons and evidence’ to reply to different challenges regarding either the under-inclusiveness or the over-inclusiveness of his definition of religion. In brief, we argue that in order to make sense of pre-theoretical intuitions about what conventionally counts as religion, Leiter faces a dilemma. Either he relies on the view that insulation from reasons and evidence is an individual epistemic attitude of believing despite the existence of discrediting evidence, in which case he cannot distinguish religion from fanatical adherence to any set of beliefs. Or he relies on a notion of insulation from reasons and evidence as a property of beliefs which, by virtue of their content, cannot be validated or invalidated by empirical evidence, in which case he cannot distinguish secular from religious conscientious commitments. As we will show, this is because the notion of ‘insulation from reasons and evidence’, as Leiter presents it, contains ambiguities arising from three sources: the subject of insulation from reasons and evidence, the meaning of the word ‘insulated’, and the meaning of ‘reasons and evidence’.

### Insulation from Reasons and Evidence: Epistemic Attitudes and Religious Doctrines

Leiter does not provide a clear definition of his notion of ‘insulation from reasons and evidence’. He rather unpacks its meaning throughout Chapter 2 while trying to separate religious commitments from other kinds of conscientious commitments and beliefs. To get the clear picture, we reconstruct his concept of insulation from reasons and evidence.

Leiter mobilizes two meanings of ‘insulation’ in order to give content to the idea of ‘insulation from reasons and evidence’. In the first sense, ‘insulation’ is a state of mind of ‘believing something notwithstanding the evidence and reasons that fail to support it or even contradict it’ (p. 39). Here, religion is just blind faith. Defining religion as faith insulated from reasons and evidence seems to make a lot of sense. In doing this, Leiter follows Timothy Macklem who argues in ‘Faith as a Secular Value’ that faith sets religion apart and explains the value of freedom of religion *qua* religion (2000).^7^ For Macklem, the term ‘religion’ refers to ‘collective participation in institutions and practices that manifest a freely given personal commitment to a particular set of beliefs, beliefs that are not based on reason alone but are held, at least in part, on the basis of faith.’^8^ Note that under this interpretation of insulation from reasons and evidence, the subject of insulation—that which is insulated from reasons and evidence—is the believer himself. Faith is a subjective epistemic attitude. For Macklem, indeed, faith is a mode of believing: it ‘describes the manner in which a particular belief or set of beliefs may be subscribed to by human beings.’^9^ Defining religion *qua* religion by referring to the notion of faith is, however, a tricky matter. For instance, Kent Greenawalt claims that faith is too vague and too common to be used in a constitutional definition of religion *qua* religion.^10^ Macklem himself recognizes that ‘faith is not always exercised in relation to beliefs that would be conventionally regarded as religious.’^11^ A concept of religion solely based on faith could thus be over-inclusive.

In the second sense, religion is insulated from reasons and evidence because it ‘neither claims support from empirical evidence nor purports to be constrained by empirical evidence’ (p. 47). Religious belief is insulated from evidence ‘not only in the sense that it does not answer to empirical evidence but also in the sense that it does not even aspire to answer such evidence’ (Ibid.). Leiter relies on this interpretation of the term ‘insulation’ when he says that religious beliefs express metaphysical propositions that purport to refer to the essence or ultimate reality of the world and transcend ordinary and empirical experience (pp. 47–49). It is not implausible to think that religious propositions are somehow insulated from certain kinds of evidence and modes of rational justification precisely because of their metaphysical content. Religion undeniably makes metaphysical claims and those claims appear to many to be insulated from empirical validation. For instance, Karl Popper famously claimed that the demarcation between science and metaphysics is that propositions belonging to the latter could not, in principle, be falsified.^12^ There is no way to test metaphysical claims; they are by their very nature insulated from empirical evidence and not disprovable by facts and experiments. In a similar (yet different) way, logical positivists, such as A.J. Ayer and Rudolf Carnap, claimed that metaphysical propositions, since they cannot be reduced to empirically observable sense-data contents, have no truth-conditions, cannot be either true or false, and are therefore non-sensical.^13^ In this second understanding, the subject of religion’s insulation from reasons and evidence is not the believer but the religious doctrines themselves. In this view, religion is insulated from reasons and evidence, not because of the subjective attitude of individuals with regard to evidence, but rather because of the very nature of religious doctrines.

The upshot is that we have two very different notions of ‘insulation from reasons and evidence’. One refers to the fact that someone believes in something notwithstanding the evidence that fails to support his belief or contradicts it. The other refers to the view that, according to the correct understanding of certain beliefs, such beliefs cannot be proven or disproven with certain kinds of evidence and reasons.

Note that these understandings of ‘insulation from reasons and evidence’ may seem to be incompatible. That is, the same belief cannot be said, without contradiction, to be insulated from reasons and evidence in both senses. For according to the first interpretation of religion’s epistemic insulation, a religious belief is insulated from reasons and evidence when the holder of such belief adopts an epistemic attitude of indifference to the reasons and evidence which actually contradicts (or fails to support) the belief in question. The individual holding belief X believes in X notwithstanding the existence of evidence Y and reason Z counting against X. This implies that belief X itself is not insulated from reasons and evidence as Y and Z can invalidate it. Yet, under the second interpretation, a belief X is itself insulated from reasons and evidence when it is such that no evidence Y or reason Z can validate or invalidate it.

One may object that there is no necessary incompatibility between the two notions of ‘insulation from reasons and evidence’. For instance, someone holding that religion is insulated from reasons and evidence could argue that the two senses of insulation nicely support one another. On this view, religion simply combines the two epistemic features: it is characterized by a *subjective* attitude of faith in ideas which cannot *objectively* be validated or refuted in the light of reasons and evidence. This objector would be immune to our inconsistency objection. However, such a person could not be the author of *Why Tolerate Religion?* Indeed—and as we’ll show further in sections “The Concept of Religion: Under-Inclusiveness and Over-Inclusiveness” and “The Epistemology of Secular and Religious Conscientious Commitments”—Leiter hesitates on the key question of whether religious beliefs are actually (objectively) insulated from reasons and evidence.

At several passages, Leiter claims that religious belief is false belief, unwarranted belief, or belief that contradicts evidence from modern science (pp. x, 39, 42). When he does so, he depicts religious belief as a subjective attitude of blind faith in ideas *despite evidence against them or despite lack of evidence*. This is the interpretation of religion’s epistemic insulation mobilized by Leiter to explain why even non-fideist and intellectualist accounts of religion are nonetheless epistemically insulated. It is also the interpretation of religion’s epistemic insulation conveyed by the definition of ‘insulation from reasons and evidence’ as ‘believing in something notwithstanding the evidence and reasons that fail to support it or even contradict it’ (p. 39), or as ‘insulation from revision in the light of evidence’ (p. 40) or as persisting in believing into something despite evidence to the contrary (pp. 41–42). Here, it appears, religious beliefs can objectively be refuted in the light of reasons and evidence.

However, when trying to distinguish religion from any form of fanatical belief, Leiter relies on the second interpretation of ‘insulation from reasons and evidence’, namely, the view that religious beliefs themselves, when properly understood, are indifferent to reasons and evidence. This is the notion of religion’s epistemic insulation which underlies the claim that religious beliefs are metaphysical in nature (p. 49) and which seems to underpin the distinction between moral judgments and religious ones from the point of view of moral realism (p. 50). Here, it is the nature of religious beliefs that they cannot be objectively refuted in the light of reasons and evidence.

### The Concept of Religion: Under-Inclusiveness and Over-Inclusiveness

We can see how the equivocal character of ‘insulation from reasons and evidence’ is significant when we turn to the argumentative strategies Leiter employs to attempt to single out religion *qua* religion.

Leiter argues that to properly single out conventional religion on the basis of its insulation from reasons and evidence, we have to locate the subject of the said insulation in the religious doctrines themselves, not in the believers themselves. Religion’s insulation from reasons and evidence is ‘a claim about the religious doctrine rather than about the typical epistemic attitudes of believers’ (p. 35).

This is necessary to distinguish religion from mere fanatical adherence to any set of beliefs. To take Leiter’s examples, the existence of a fanatical defender of the theory of gravity who ‘does not even worry about how evidence of the expansion of the universe squares with [this] theory’ does not make the theory of gravity a religion (p. 35). Similarly, Marxism is not a religion, even if some Marxists (‘committed communists’, p. 40) disregard—or do not bother examining—empirical evidence. Correctly understood, Marxism is a theory of historical change which is supposed to answer the same standards of evidence and rational justification as any other scientific theory (p. 38). By contrast, we understand that if religion is to be distinct from the theory of gravity and classical Marxism, it is because it stands in a different relation to those standards of evidence and rational justification; religious doctrines themselves, not believers in their individual epistemic attitudes, are not supposed to be responsive to such evidence.

So far, so good. Yet, things get messy when we turn our attention to ambiguities concerning the kinds of reasons and evidence from which religion is allegedly insulated. Leiter starts with a very broad notion of ‘reasons and evidence’ and he asserts that religion is insulated from reasons and evidence ‘as these are understood in other domains concerned with knowledge of the world’ and from ‘ordinary standards of evidence and rational justification, the ones we employ in both common sense and in science’ (p. 34), thereby only excluding divine revelation from ‘reasons and evidence’.

The problem with this is that it provides an under-inclusive definition of religion, one which rules out any belief in the existence of God supported by philosophical modes of rational enquiry abiding to the laws of logic and sound inference. Examples include St-Anselm and Descartes’s ontological argument (it is inconceivable that God lacks the predicate of existence),^14^ Kant’s transcendental argument from morality (God’s existence is necessary for the existence of objective moral truths and of moral agents),^15^ Aquinas’s five arguments for the existence of God based on natural reason,^16^ Ibn Rushd’s (Averroes’s) teleological argument (there must be an intelligent creator, since the working of the whole world is fine-tuned to serve the purpose of humanity),^17^ and Aristotle’s cosmological argument (there must be a first uncaused or *sui*-*generis* cause for the existence of everything that exists).^18^ Perhaps, of course, those are bad and fallacious arguments. Kant himself, and many others, raised philosophical objections against the ontological, teleological and cosmological arguments.^19^ Yet, when one asserts that those kinds of arguments supporting some beliefs expressing religious propositions are erroneous arguments, one is bound to recognize, *ipso facto*, that these (religious) beliefs are not insulated from rational enquiry; they can be disproved by such a rational and philosophical enquiry.^20^

Leiter claims that those rationalist interpretations of conventional religious belief are the fruit of mere ‘post hoc rationalization’ and that those beliefs, although purportedly justified by rational means, are still insulated from revision in the light of evidence, because, according to him: ‘it never turns out that the fundamental beliefs are revised in light of new evidence’ (p. 40). In other words, Leiter’s strategy to avoid the conclusion that rationalist interpretations of religion are not religious, is to claim that those who make such rational arguments in support of their religious belief are still, at the level of their subjective epistemic attitudes, insulating themselves from reasons and evidence.

Nothing in what Leiter says to present non-fideist accounts of religion as being insulated from reasons and evidence implies that the religious beliefs and doctrines which are purportedly justified by rational philosophical arguments are themselves insulated from reasons and evidence. Quite to the contrary, as noted earlier, the fact that so many have provided counter-arguments and objections to rational arguments for the existence of God, and that Leiter himself insinuates that they can or should be revised in light of new evidence, implies that such beliefs are themselves not insulated from rational objections and from discrediting evidence. Leiter is thus only able to incorporate rationalistic interpretation of religious belief into the concept of religion at the price of abandoning the concept of ‘insulation from reasons and evidence’ which allows him to distinguish religious belief from fanatical adherence to any kind of belief.

There is one way in which Leiter could avoid the problem of under-inclusiveness of the concept of religion while locating insulation from reasons and evidence at the level of religious beliefs themselves. He could narrow his understanding of the relevant kinds of reasons and evidence from which conventional religion is insulated so as to exclude the kind of philosophical justification usually alluded to in rational arguments in support of religious belief. Leiter veers towards such a narrowing strategy in later parts of his book. Although he starts with a very broad interpretation of ‘insulation from reasons and evidence’ (encompassing standards accepted in all forms of knowledge other than religion), he moves towards the view that religion is only insulated from hard empirical evidence and standards of justification accepted in science (pp. 38, 57) and, sometimes, only from empirical evidence (p. 47). Thus perhaps the demarcating feature of conventional religious beliefs is that they are, when properly understood, insulated from *naturalist* standards of justification. This seems to fit quite well with the claim that religious beliefs are insulated from reasons and evidence because they express metaphysical propositions which transcend the world of ordinary experience.

Yet, as Leiter remarks, some religious beliefs are purportedly supported by one type of empirical evidence, namely by past testimonial evidence recorded by written sources. For instance, the Gospels provide testimony of the existence of Jesus Christ, of his performance of miracles and of his resurrection. Leiter claims, however, that since such testimonial evidence contradicts massive amounts of different testimonial evidence and of the evidence from biology and physiology, it should be given no credence so that ‘devout Catholics who still persist in believing in the resurrection of Christ hold that belief insulated from reasons and evidence’ (p. 42). Once again, what Leiter demonstrates here is that religious believers are themselves insulated from reasons and evidence in the sense that they are blinded by faith and continue to believe in something notwithstanding the evidence against it. He does not show that the beliefs in question are themselves insulated from reasons and evidence. Quite to the contrary, those beliefs can be shown to be false by appealing to ‘massive amount of testimonial evidence, as well as the evidence of physiology and biology’ (p. 42). They are therefore not insulated from empirical evidence and standards of rational justification as these are understood in the natural sciences.

### The Epistemology of Secular and Religious Conscientious Commitments

We should, however, refrain from quickly jumping to the conclusion that insulation from reasons and evidence cannot be the distinctive feature of religion. Perhaps the previous objection draws too much on particular examples and only applies to some religious beliefs (those allegedly supported by testimonial evidence but in fact refuted by more evidence). Leiter may still claim that these beliefs are not the ones that are crucial for the singling out of religion as such, as these are not beliefs issuing in categorical demands upon the believers. And he may claim that it is those categorical beliefs, because they express metaphysical propositions and are insulated from empirical evidence, that are distinctive of religion.

Leiter does not show that religious doctrines contain such central beliefs (that issue in categorical demands and are insulated from reasons and evidence in the relevant way) yet this is quite a plausible assumption. For instance, certain strands of Buddhism assert that the act of eating meat spreads fear among living creatures and goes against the virtue of compassion and that it should therefore be prohibited to eat sentient beings; members of the Religious Society of Friends, the Quakers, believe that all wars and outward fighting proceed from men’s lust and derive a categorical commitment to pacifism from this belief; 21 members of the Hutterian Brethren Church of Wilson believe that the second commandment (‘You shall not make for yourself an idol’) is a divine prescription which forbids them from having photographs taken of them.^22^

Such beliefs issuing in categorical demands seem to be widespread in conventional religions and are arguably insulated from empirical evidence and standards of justification found in natural sciences. Have we thus found a way of singling out religion as such by using an unambiguous notion of ‘insulation from reasons and evidence’? Hardly: this concept of religion makes religious claims of conscience very similar to the categorical demands of the secular moral conscience placed upon non-believers. Indeed, a secular belief that all human beings are equal in dignity, and that this generates certain categorical demands (not to kill other human beings, not to discriminate on the basis of race, gender and religious belief) seems to be as much insulated from empirical evidence and standards of justification used in the natural sciences as the religious beliefs mentioned in the previous paragraph. A secular belief that violence and the use of weapons to kill other human beings is always wrong generates a commitment to pacifism that can be as much insulated from empirical evidence as the commitment of the pacifist Quakers. And a secular belief that life has intrinsic value can ground an ethical commitment to vegetarianism while being just as impossible to prove with empirical evidence and the tools of modern science as the vegetarian Buddhist’s convictions.^23^

Leiter is reluctant to analogize religious prescriptions with secular morality in such a way. He claims that religious categorical demands are distinct from moral commands in general (p. 49–52). This is surprising since he also asserts that claims of conscience, all claims of conscience, including religious ones, are moral imperatives: ‘[a] claim of conscience is, after all, a claim about what one *must* do, no matter what—not as a matter of crass self-interest but because it is a kind of moral imperative central to one’s integrity as a person, to the meaning of one’s life’ (p. 95).

If this is so, why does Leiter insist in viewing religious claims of conscience, making categorical demands about what one must do, as being so different from the claims of secular morality? Leiter’s strategy is to analyze religious propositions and moral propositions from the point of view of meta-ethics, that is, from the point of view of the semantics of moral propositions (what we take judgements of right and wrong, good and bad, to be about) and of the metaphysics of such propositions (whether or not we take moral facts to exist). Leiter remains agnostic with regard to those meta-ethical questions and attempts to show that on any possible account of the semantics and metaphysics of moral claims, it appears that morality and religion differ in light of the insulation from reasons and evidence criterion. As we demonstrate in the following, however, Leiter puts forward a brave but ultimately too sweeping analysis of complex questions of meta-ethics and epistemology of religion.

Leiter proceeds by arguing that either on realist accounts of morality, such as Peter Railton’s or Richard Boyd’s, or on antirealist accounts of morality, such as expressivism, religion differs from morality on the basis of the former’s insulation from empirical evidence and scientific methods.

Religious claims of conscience, according to Leiter, fit neither cognitivist realism nor non-cognitivist antirealism (p. 50–51). Moral realism asserts that moral propositions refer to moral facts about the world. Therefore, argues Leiter, from the realist’s perspective, moral propositions are not insulated from reasons and evidence as is religion. On the other hand, antirealism asserts that moral sentences do not express beliefs about the world: they merely express subjective attitudes of approval and disapproval and therefore lack truth-conditions. Such a non-cognitivist and expressivist stance implies that moral judgements are insulated from beliefs and evidence and cannot be true or false. Leiter asserts that religious claims of conscience do not lack truth-conditions in this way since they purport to express something about the world and thus ‘in principle, could be answerable to reasons and evidence, but are nonetheless taken to be insulated from them’ (p. 51).

This argument is fallacious since it presents us with a false dichotomy and misrepresents both realism and non-cognitivism. First, Leiter relies on a false dichotomy. Several strands of moral realism view moral propositions as referring to non-natural moral facts such that the truth and falsity of moral judgements cannot be established by appealing to empirical evidence and scientific methods. For instance, G.E. Moore developed an intuitionist form of moral realism asserting that moral facts are not empirically observable and detectable with the tools of the natural sciences and that humans have a faculty of intuition allowing us to directly observe non-natural moral properties, such as ‘goodness’, which are non-reducible and non-identical to natural properties.^24^ According to this cognitivist understanding of morality, moral beliefs are insulated from empirical evidence and rational justifications as understood in the natural sciences. Other forms of non-naturalistic realism also assert that, although there are moral facts, those are non-natural facts which are not the subject-matter of empirical sciences. For instance, John McDowell, a moral realist, views moral facts as facts about the reasons that we have to act in certain ways and claims that we can be brought to recognize via proper upbringing.^25^

Leiter could of course maintain that these non-naturalist accounts of moral realism are implausible. However, his argument would thereby lose its meta-ethical agnosticism and become hostage to a controversial meta-ethical position. Even if he were ready to bite this bullet and to defend the truth of naturalist moral realism, Leiter would still have to explain how certain core elements of the versions of naturalist moral realism he refers to are not, at least partially, insulated from empirical evidence and scientific demonstration. For instance, for Railton, whom Leiter takes as representative of naturalist moral realism, a person’s good is determined from an individual idealized point of view of full information and consists in ‘what [this person] would want himself to want, or to pursue, were he to contemplate his present situation from a standpoint fully and vividly informed about himself and his circumstances, and entirely free of cognitive error or lapses of instrumental rationality.’^26^ Moral rightness, in turn, is viewed by Railton as what is rational from an idealized social point of view so that what is morally right is ‘what would be rationally approved of were the interests of all potentially affected individuals counted equally under circumstances of full and vivid information.’^27^ It is difficult to imagine how the counter-factual judgements that would be obtained in such idealized situations could be entirely based on empirical evidence and the methods of the natural sciences. Rather, determining the parameters and outcomes of idealized individual and social rational decision-making procedures seems to invite at least some speculative philosophical reasoning roughly akin to the one at play in the above-mentioned rational arguments in support of religious beliefs and their objections.

In brief, Leiter exaggerates the extent to which, under the cognitivist umbrella, moral beliefs answer to empirical evidence and scientific proofs. If that is the case, it is difficult to distinguish moral and religious beliefs in the way that he wants to. Now, how does Leiter conceives the relation between secular morality and religion from the antirealist standpoint? Leiter surprisingly asserts that from an expressivist point of view all moral propositions differ from religious ones, precisely *because* they are insulated from reasons and evidence as understood from a naturalist point of view. For expressivists, moral statements are not apt to be true or false because they merely express subjective feelings, emotions or attitudes of approval or disapproval.

Leiter’s strategy is thus to claim that religion is insulated from evidence in a different way than moral propositions expressing subjective feelings: ‘religious judgements *do express beliefs*’. They could ‘in principle, … be answerable to reasons and evidence, but are nonetheless taken to be insulated from them’ (p. 51). This strategy has a number of flaws. To begin with, it makes Leiter, once again, rely on the view that religion’s insulation from reasons and evidence has to do with believers’ epistemic attitudes (‘they are *taken* to be insulated’) rather than with the beliefs themselves, since those are presented as being in principle not insulated from reasons and evidence. The problem with this, as we saw, is that this view makes religion indistinguishable from any fanatical adherence to any set of beliefs.

Furthermore, why would expressivists interpret the value judgements made by religious individuals as referring to external facts rather than merely expressing subjective feelings? An expressivist views all propositions taking a form like ‘X must do Y because of Z’, ‘Y is good’, ‘Y is good because of Z’ as propositions expressing the subjective feelings of the speaker rather than purporting to describe some objective moral state of affairs. This is so regardless of the religious character of the proposition (of Z in the example above). Why would an expressivist accept that the secular vegetarian stating his convictions only expresses an attitude of disapproval towards meat-eating but that the Buddhist vegetarian purports to describe some state of affairs when stating his own commitment? As Dworkin explains, religions do have a ‘science department’, a set of beliefs purporting to describe how the world really is. Yet, they also have, as Dworkin maintains, a ‘value department’: they contain beliefs which are moral in character as they concern what individuals must do, what has intrinsic value, and so on.^28^ For an expressivist, propositions pertaining to the value department of religions are just as much expressions of subjective attitudes insulated from empirical evidence as non-religious moral propositions. There is nothing special about religion here. Moreover, recall that for the most prominent theorist of expressivism, A.J. Ayer, even descriptive or ‘scientific’ religious propositions, such as ‘God exists’ are non-sensical, to the same extent that moral ones are: they have no truth conditions and would thus be, in Leiter’s terminology, insulated from reasons and evidence.^29^ So it seems that in both major accounts of meta-ethics, Leiter fails to distinguish religious claims of conscience from secular morality. The idea that religion is insulated from reasons and evidence cannot be the demarcating feature which singles out religion *vis*-*à*-*vis* secular morality.

In sum, it seems that Leiter either fails to distinguish religion from fanatical adherence to any kind of belief (when he views insulation from reasons and evidence as a subjective attitude) or he cannot distinguish religion from secular moral doctrines (when he relies on the notion of insulation from empirical evidence and naturalist standards of justification as a property of religious doctrines). This is a serious shortcoming in Leiter’s overall approach to the problem of singling out religion. Leiter attempts to solve this problem by asking whether there are good reasons to tolerate religion, as such, given its defining features. But as the defining features he provides are not specific to religion, they do not help us answer the question as to whether we should tolerate religion. Leiter, in particular, has not explained why we should tolerate secular conscience, given that conscience shares the very features he singles out as specifically religious.

### Existential Consolation and Secular Worldviews

Leiter may claim that our analysis has left out an important feature of religious belief which distinguishes religion from other worldviews and systems of thought. He may claim that what ultimately distinguishes religious beliefs from morality in general and from secular worldviews and political ideologies is that religion offers existential consolation: it ‘render[s] intelligible and tolerable the basic existential facts about human life, such as suffering and death’ (p. 52). This is unconvincing.

First, Leiter himself recognizes that non-religious philosophical reflection, meditation and therapeutic treatment provide existential consolation (p. 62). He claims that those forms of existential consolation are not insulated from reasons and evidence, without specifying what kind of epistemic insulation is at play here. Much philosophical reflection is insulated from standards of evidence as narrowly defined in the natural sciences, for example. Furthermore, philosophers since the Ancient period have produced countless ethical systems and worldviews aiming at providing existential consolation. Perhaps, Leiter would argue that these were all religious philosophies. So be it. Yet, more recently, atheist philosophers concerned with the relation between secular philosophy and religion have tried to explain how non-theistic philosophical doctrines could provide some form of existential consolation. For instance, Thomas Nagel explains that a few secular options (humanism, Nietzschean Darwinism, and a revised form of Platonism) could provide at least a partial answer to the ‘cosmic question’ of how to live in harmony with the universe as a whole and not just as an individual in it.^30^ Ronald Dworkin, in the very last moments of his life, wrote that we can make sense of immortality by viewing one’s life as an objectively and timelessly valuable achievement.^31^ Perhaps those secular attempts are vain and ultimately fail to provide existential consolation. However, this failure cannot, from Leiter’s point of view, be the site of the distinction between religious beliefs and secular philosophical worldviews since he understands the former, as we have seen, as being either false or improvable. Claiming that secular philosophy’s actual failure to provide existential consolation distinguishes it from religion as a provider of existential consolation would put Leiter at odds with his own sceptical treatment of religion.

To recap, many non-religious doctrines contain beliefs that (1) are insulated from reasons and evidence, (2) make categorical demands and (3) provide existential consolation. Leiter’s definition of religion proves to be over-inclusive and does not allow him to single out religion. As a result, he has not explained why secular conscience, when it displays the same problematically epistemic features as religion, deserves respect.

## Toleration, Respect and Exemptions

Let us now turn to what Leiter exactly means by ‘tolerating’ and ‘respecting’ religion and conscience. Leiter’s argument can be summarized as follows:

1. Existing theories of principled toleration cannot justify tolerating religion *qua* religion (Chapter 3).
2. Religion *qua* religion does not deserve special respect—i.e., ‘appraisal’ respect (Chapter 4).
3. Therefore, there are no good reasons for granting exemptions to religion *qua* religion. Exemptions, if they are justifiable at all, should be available for both the religious and the non-religious conscience.
4. Moreover, there are no good reasons to grant exemptions for burden-shifting claims of conscience (religious or otherwise).

Although we are sympathetic to Leiter’s conclusion that the special legal status of religion with regard to exemptions to the law is problematic (3), we do not think that this conclusion follows from the argument he develops in his book. For Leiter’s conclusion (3) to follow, premises (1) and (2) need to be complemented by another premise, namely, that:

(2bis) legal exemptions can only be justified on the basis of principled toleration or on the basis of appraisal respect.

Yet, as we will argue in this section, this underlying premise (2bis) is mistaken on two grounds. *First, there is no straightforward connection between principled toleration and practices of exemptions. Second, such practices need not be justified by an attitude of appraisal respect*. In addition, we will argue that Leiter’s version of the No-exemption (except for non-burden-shifting claims of conscience) approach (4) is morally problematic as it does not derive from a principled argument.

### Theories of Toleration, Theories of Exemptions, and Respect-Based Theories of Exemptions

Leiter’s argument against the special status of religion stages a nexus of relationships between the notions of toleration, respect and legal exemptions. Properly to assess this argument, we first make a few preliminary distinctions and observations.

First, toleration is conceptually linked to a negative attitude of disapproval or dislike towards the practice that is tolerated. The ‘circumstances of toleration’ imply that individuals or groups espouse practices or beliefs which are disliked or disapproved of by the groups who have the power to interfere with those beliefs and practices.^32^ As Susan Mendus remarks: ‘[w]e cannot, properly speaking, be said to tolerate things which we welcome, or endorse, or find attractive.’^33^ Thus, tolerating others who are different from us is distinct from respecting them on the basis of approval or admiration of what makes them different.^34^ Such a positive attitude of respect and support for others’ differences is often seen as going ‘beyond toleration’^35^ Leiter embraces this distinction. He claims that toleration is hardly distinguishable from an attitude based on ‘recognition respect’, which simply requires one not to violate basic moral requirements and general moral obligations owed to others as persons. He then contrasts this ‘negative’ form of respect (not infringing others’ rights) from a more ‘positive’ form of respect, which he calls ‘appraisal respect’, and which involves expressing admiration and high esteem of someone because of his or her valuable achievements (pp. 69–72). Toleration and appraisal respect are thus two distinct attitudes. It is in light of those two attitudes that Leiter discusses the special legal status of religion *vis*-*à*-*vis* legal exemptions, investigating whether one or the other provides grounds for treating religion as the unique recipient of legal exemptions.

Second, we must distinguish between a theory of toleration and a theory of legal exemptions. Toleration is associated with the separation of church and state as it emerged in the aftermath of the European religious wars of the sixteenth and seventeenth centuries. Toleration is based on the creation of a protected private sphere of non-interference in which the individual is free to adopt his or her own religious or moral views; it shields certain areas of private life from political intervention.^36^ John Locke conceived of toleration in this way: he famously argued that political power should be limited to the protection of ‘civil interests’, namely the preservation of ‘liberty, health, and indolency of body; and the possession of outward things’, and that the power of the state should not extend to ‘the salvation of souls.’^37^ Note that, for Locke, although political authority should never be exercised for the pursuit of spiritual goals (for instance, in order to convert religious dissidents and suppress their views), the government is entitled to restrict the free exercise of religion when this is necessary to further civil interests. Here, toleration only requires non-interference in the private sphere and nothing like a right to manifest one’s religious beliefs in the public realm. It does not require protecting religious freedom by granting legal exemptions from laws pursuing valid civic goals. As Locke claims: ‘the private judgement of any person concerning a law enacted in political matters, for the public good, does not take away the obligation of that law, nor deserve a dispensation.’^38^

Toleration requires governments to refrain from interfering with certain unpopular practices or beliefs on the ground that these practices or beliefs are disliked or disapproved by the dominant group.^39^ Toleration is nonetheless compatible with certain forms of interference that are not justified on the basis that some practices and beliefs are false, morally inferior, or repugnant. Thus, toleration does not preclude interference justified by an appeal to some neutral (non-perfectionist) grounds such as, for instance, the necessity to restrict the liberty of individuals who want to exercise their liberty in harmful ways, leading to the negation of others’ fundamental rights. There is also no contradiction in claiming that toleration is compatible with allowing some religious practices to be restricted because this is required to pursue some legitimate and neutral collective goal, such as ensuring security, health and safety.

A theory of legal exemptions, on the other hand, goes further than this. It must explain why it is sometimes permissible, or even required by justice, for a government to allow some individuals or groups not to comply with certain laws of general applicability, which are neutrally justified and pursue legitimate collective objectives.^40^ Exemptionism is more demanding than toleration as it is usually understood. The latter forbids all interferences based on non-neutral reasons and the former forbids some interferences based on neutral justifications. A theory of legal exemptions is premised on the view that sometimes toleration as non-interference (or, more precisely, no interference for non-neutral reasons) is insufficient to adequately protect the political values of the liberal state. A theory of legal exemptions explains, for instance, why granting Sikhs an exemption to helmet laws may be required or desirable, which is left unaddressed by standard accounts of toleration. Indeed, standard accounts of toleration are preoccupied with cases of direct discrimination or religious persecution in which certain groups are targeted by laws or practices imposing a special burden upon them because they are disliked by those in power. They are not preoccupied with cases of indirect discrimination, that is, unforeseen and unintended discrimination resulting from the application of neutrally justified laws and regulations. In general, theories of exemptions explain that liberty of religion and of conscience needs more robust protection than toleration as non-interference because neutrally justified laws sometimes impose an unfair burden upon some individuals.^41^

Third, the view that religion should be treated as special under the law by being the sole recipient of exemptions to the law can also be based on the notion of appraisal respect for religion. Some theories of religious freedom are (appraisal) respect-based. Those theories assert that religious freedom deserves special constitutional protection because religious practices and beliefs are worthy of esteem and admiration. In this view, religious freedom protects the intrinsic value of religious devotion or the instrumental value of religion, that is, its distinctive capacity to bring about desirable outcomes. Respect-based theories of religious freedom claim that religious practices and beliefs ought to be protected because they are a necessary constituent of the human good or because religious devotion is socially useful. Michael Sandel puts forth such a theory of religious freedom. He claims that ‘[t]he case for according special protection to the free exercise of religion presupposes that religious belief, as characteristically practiced in a particular society, produces ways of being and acting that are worthy of honor and appreciation—either because they are admirable in themselves or because they foster qualities of character that make good citizens.’^42^ Starting from such an (appraisal) respect-based theory of religious freedom, one could argue that the purpose of exemptions to the law is to protect or promote what is good and valuable in religion.

With those preliminary observations in mind, let us now turn to Leiter’s argument. In order to assess whether there are good reasons to grant religion a special legal status, Leiter turns to three principled arguments for toleration and asks whether under each of those three accounts, we have good reasons to tolerate religion as such (Chapter 3). He addresses a Rawlsian contractualist argument, a Millian epistemic argument and a utilitarian argument. In what follows, we argue that this strategy wrongly assumes that there is a direct logical route between toleration and practices of exemption. We show that the first two arguments for toleration are not designed to justify legal exemptions in the first place (2.2) and the third one, while it can be formulated as an argument for religious exemptions, turns out to be an (appraisal) respect-based argument. As a result, Leiter ignores those theories which justify legal exemptions not because they are required by toleration, nor because religion is especially valuable (including on utilitarian grounds), but on other grounds—such as equality or fairness (2.3). In sum, Leiter has not looked in the right place, and has therefore failed to take seriously the most convincing arguments in favour of legal exemptions. We develop this line of criticism in the next two sections before turning to Leiter’s view regarding the No-exemption approach.

### Toleration and Exemptions

In order to answer the question as to whether special religious exemptions can be justified, Leiter asks whether classical theories of toleration single out religion as special. Yet—we argue—this confuses two distinct inquiries, and there is no reason to think that a negative answer to the latter will generate a negative answer to the former.

Of the three arguments for toleration that Leiter discusses, the first two turn out to be unconnected with practices of exemptions, and the third is an argument based on appraisal respect. So none is fit for Leiter’s purpose; but there is nothing surprising about this. Leiter is right to point out that the special status given to religion in most Western legal system raises important philosophical questions. Leiter is also right to examine the grounds of religious liberty in order to determine whether non-religious commitments should benefit from similar constitutional protection. Yet, he errs when he assumes that classical theories of toleration can provide the grounds for practices of religious exemptions.

The Rawlsian contractualist argument asserts that the contracting parties of the original position would choose the principle of equal liberty since they ‘cannot take chances with their liberty by permitting dominant religious or moral doctrine to persecute or to suppress others if it wishes.’^43^ The Millian epistemic argument claims that tolerating the expression of a diversity of opinions in society is both necessary to attain the truth and to provide experiences in living which allow individuals to make more informed choices about how to live their lives.^44^

One thing is immediately clear. These two arguments—Rawlsian and Millian—are not arguments for religious exemptions; they are arguments for toleration as non-interference with private beliefs and practices—very much in the spirit of the Lockean conception of toleration outlined above. So it is unsurprising that they would not permit singling out religion for special legal protection. Rawls argues that his principle of equal liberty of conscience only requires governments to refrain from imposing a religious orthodoxy and to refrain from imposing the conception of the good they hold to be true and morally superior. In *A Theory of Justice*, he claims that equal liberty of conscience requires rejecting ‘the notion of a confessional state’ as well as that ‘of the omnipotent laicist state’, which are both based on the view that the state has the authority to suppress religious and philosophical doctrines it deems illegitimate.^45^ Similarly, Mill’s epistemic argument for tolerating diverse lifestyles only requires that freedom of expression be respected and that individuals be able to pursue their life plans in the private realm. The only conclusion that Mill draws is that one’s liberty should only be restricted in order to protect others against potential harms. Mill famously opposed coercing others for paternalistic reasons (for instance, to save them from their false beliefs) or for moralistic reasons (because their behaviour offends and disgusts us). Thus both Rawls’s and Mill’s arguments fall squarely into the frame of toleration as we defined it: forbidding interference for non-neutral reasons.

In sum, Leiter focuses on arguments that were intended neither to justify exemptions from the law, nor to single out religion as such. Rawls and Mill have provided the canonical liberal arguments for what we could call equal moral freedom—equality before the law for all individuals regardless of their religious or non-religious beliefs. There is no direct logical route between toleration as equal moral freedom and special exemptions from the law on religious grounds. At best, the argument for exemptions could be a derivative argument. But we should not expect to find it in the canonical texts themselves.^46^

### Respect and Exemptions

Leiter, however, explores another principled argument for toleration. As we will see, this argument can justify practices of exemptions, yet it is not an argument for tolerating practices which are disliked but, rather an argument for supporting practices we should approve of and view in a positive light. In sum, it is closer to appraisal respect than it is to recognition respect and toleration.

Leiter considers whether, from a utilitarian perspective, it would make sense to say that religious beliefs are especially conducive to happiness or have, *qua* religious beliefs, a special utility-enhancing function (pp. 59–61). Leiter asks whether this argument can ‘provide a utilitarian rationale for singling out matters of religious conscience for special protection’ (p. 61). We could thus construe a utilitarian argument asserting that allowing religious exemptions can be justified if it in fact produces more good than refusing those exemptions would. Leiter considers, and rejects, this argument. In particular, he refuses to ‘bite the speculative bullet’ and to claim that the potential of religious beliefs to produce happiness (by providing existential consolation) is greater than the potential for harm of religious beliefs. This potential for harm is in fact significantly high since, as Leiter asserts, the combination of categoricity and insulation from reasons and evidence is a ‘potentially harmful brew’ (p. 62).

So the only possible ground for exemptions to the law that Leiter (briefly) considers is the utilitarian argument for principled toleration. For him, it is only if religious beliefs and practices are conducive of distinctively good outcomes that there is place for special legal protection. As Leiter claims that conductivity to good outcomes is a source of appraisal respect, a basis for positive valuation and for being the object of esteem (p. 85), it appears that he only conceives of exemptions as being justifiable on the basis of ‘appraisal respect’. Leiter does not take seriously the possibility that the justification of religious exemptions can be based on something else than positive endorsement of religion. No wonder he asserts that carving out special legal protection for religion as such is tantamount to encouraging religion (p. 63).

Consequently, Leiter’s treatment of legal exemptions contains a blind spot. Leiter never discusses any argument purporting to justify such exemptions, except one argument, the utilitarian one, which requires adopting a positive valuation of religious belief in general. Paradoxically, he then draws closer to those very theories of positive valuation of religion—Sandel’s and Modood’s in our examples—against which *Why Tolerate Religion?* is targeted. But this also blinds Leiter to the fact that, in both jurisprudence and the political theory literature, exemptions are justified by appealing to basic liberal ideals of freedom, equality and inclusion, rather than a positive valuation of religion *per se.* 47

For example, it has been argued that exemptions are justified because they are required to give members of religious and cultural minorities ‘the same opportunity as enjoyed by the majority of citizens to combine their (reasonable) cultural or religious pursuits with basic civic opportunities like employment and education’; 48 or because they are required to address the loss of integrity and alienation resulting from acting against the grain of one’s conscience^49^; or because, in a society dominated by a religious majority, equality and non-discrimination sometimes require granting exemptions to religious minorities.^50^ These arguments reflect the jurisprudence of most Western countries in which legal exemptions, when they are granted, are justified on the basis of the protection of anti-discrimination rights and on the right to freedom of religion and of conscience. These arguments might be flawed. And they might be flawed for exactly the reason that Leiter identifies, namely, that they do not explain what it is about religion that makes it a basic and special right. But Leiter makes it too easy for himself by only considering theories of toleration and respect-based theories of exemptions, thereby neglecting the most promising approaches to the ‘exemption puzzle’.

### Justice and Burden-Shifting Exemptions

After having examined arguments for principled toleration, Leiter concludes that there is no good reason to single out religion for special protection in the form of legal exemptions. Yet he has not really addressed the question of the legitimacy of exemptions in the first place. The way he deals with the issue, towards the end of the book, is by asking whether existing exemptions should be extended to non-religious commitments—in particular, secular convictions of conscience.

Leiter opposes such an extension, and instead defends a version of Brian Barry’s ‘No-exemptions approach’.^51^ In Leiter’s view, no exemptions should be allowed, neither for secular nor for religious commitments, with the exception of exemptions that do not impose any burden or cost on those who are not exempted. Leiter highlights the practical difficulties associated with expanding the regimes of religious exemptions to all claims of conscience: such a universalization would generalize and constitutionalize the right not to comply with the law, making it harder to enforce the law; moreover, it is difficult for courts to assess the authenticity of individual claims of conscience (p. 94–96).

Putting aside those important practical concerns, Leiter raises one principled objection to practices of exemptions; namely, those practices ‘often impose burdens on those who have no claim of exemption’ (p. 99). For instance, as he points out, in periods of forced induction into the army, when someone is exempted from military service on conscientious grounds, the burden of taking up arms must be shouldered by another citizen who has no claim to be a conscientious objector. Leiter does not cite many examples of burden-shifting exemptions to the law, but several come to mind. For instance, one may argue that, in countries where health services are publicly subsidized, exempting some religious groups from drug laws may increase the social costs related to the treatment of drug addiction, and perhaps exemptions from laws requiring the wearing of helmets for motorcycle drivers and for construction workers may increase public health expenditures related to head injury treatments.

Such considerations are plausible. Yet they seem to be dependent on a prior theory of fairness, equality, and justice—of the kind that, as we pointed out, Leiter fails to consider. Still, we may ask: on which *implicit* theory of justice, of fair distribution of the burdens and benefits of social cooperation, does Leiter rely when he claims that burden-shifting exemptions impose an unjust burden on those who have no claim to being exempted from the law? As he provides no clues to answer this question, it seems that Leiter’s argument amounts to saying that *any shift* in the existing distribution of burdens is unfair by the sheer fact of introducing a modification into the existing pattern of distribution of burdens of social cooperation. This assumes that currently existing laws and institutional arrangements are already fair and constitute an appropriate baseline against which demands for exemptions can be evaluated from a moral point of view. There is no reason, however, to assume that currently existing laws and administrative regulations provide an impartial standpoint to assess whether demands for exemptions impose unfair burdens on the rest of society. We need an independent criterion, one not tied to existing institutions, laws, and practices in order to make valid judgments about the fairness of the distribution of the burdens placed by coercive laws upon citizens embracing different religious and philosophical views.

It is not our aim here is to provide such a criterion. We simply want to highlight that Leiter’s endorsement of the rule of ‘No-exemption except for non-burden-shifting exemptions’ commits what we call the ‘status-quo neutrality’ fallacy. In other words, Leiter embraces a theoretical position, which takes the existing distribution of burdens and benefits in society for granted and which fails to provide an impartial baseline from which current claims about inequalities or unjust treatment can be normatively assessed.^52^ The objection to all burden-shifting exemptions simply assumes that the actual distribution of burdens under a regime of universally applicable laws with no possibility for exemptions (except for non-burden-shifting ones) is already fair and necessarily unproblematic. Adopting this viewpoint, Leiter proves incapable of thinking about regimes of legal exemptions to the law for conscientious citizens through the critical lenses of a conception of justice. As a result, he fails to develop a convincing principled argument against practices of exemptions to the law.

## Conclusion: Why Tolerate Conscience?

The title of this article is an amphibology. By it, we want to ask Leiter two questions, slightly different from the one he asks in the title of his book, *Why Tolerate religion?* First, Leiter maintains that while there is nothing special about religion, conscience should be the object of toleration. However, in section “Religion and Conscience”, we argued that Leiter is hard-pressed to distinguish between religious and secular claims of conscience. But then, why should secular conscience be tolerated when it exhibits exactly the same features as religion (namely, categoricity, existential consolation and insulation from empirical evidence)? Although he spends much time and effort defining religion, Leiter does not tell us what exactly in conscience, whether it is secular or religious, is worthy of toleration. Should we tolerate conscience because conscientious beliefs make categorical demands, because they are central to individuals’ identities, because people are happier when they are able to live in accordance with their consciences, or because of something else? In other words, why tolerate *conscience*? Second, in section “Toleration, Respect and Exemptions”, we contrasted classical theories of toleration as non-interference with individuals’ private choices and theories of legal exemptions. We argued that, by addressing the issues raised by special religious exemptions only through the lenses of toleration (and appraisal respect), Leiter misses the point of exemptions. His approach seems to be question-begging. Of course principled toleration does not offer reasons to single out religion as the sole recipient of legal exemptions: it does not provide a justification for exemptions in the first place. Why does Leiter assume that toleration is the most salient political concept to discuss the legitimacy of practices of legal exemptions for religious and secular claims of conscience? Indeed, why *tolerate* conscience?

## Footnotes

1 Leiter (2013).

2 For those two dimensions of the special legal status of religion (protection and disability), see Schwartzman (2012).

3 There are exceptions to this general trend. For instance, as is well known, the United States' Supreme Court, both in the *Seeger* and *Welsh* decisions, decided that individuals could be exempted from forced induction into the Army even though their pacifists convictions were not religious. The Court's rationale was that the secular convictions of the plaintiffs played a similar role in their lives as the one usually played by religious convictions in the lives of believers. See United States v. Seeger 380 U.S. 163 (1965) and Welsh v. United States 398 U.S. 333 (1970).

4 Multani v. Commission scolaire Marguerite-Bourgeoys, (2006) 1 S.C.R. 256, 2006 SCC at para. 74.

5 See for instance, Greenawalt (1984), Laycock (1996), McConnell (2000), Koppelman (2006), Eisgruber and Sager (2007), Nussbaum (2008), Greene (2009), Maclure and Taylor (2011), Schwartzman (2012), Dworkin (2013), Laborde (2014).

6 Those are exemptions that impose burdens on third parties who do not benefit from similar exemptions to the law.

7 Macklem (2000).

8 Macklem (2000, p. 27).

9 Macklem (2000, p. 33). Many accounts of epistemology of religion understand faith as a subjective epistemic attitude. See for instance, Audi (2011, pp. 54, 59) and Swinburne (1981, p. 118).

10 Greenawalt (1984, pp. 768–769).

11 Macklem (2000, pp. 27–28).

12 Popper (2002, pp. 345–346).

13 Ayer (1952). Carnap embraced a more ‘liberal’ and permissive criterion of meaning (confirmability). See Carnap (1953).

14 Descartes (1993, pp. 24–35, 42–47), St-Anselm (1965, pp. 3–6).

15 Kant (1943, pp. 451–460).

16 Aquinas *(* 1947, pp. 11–14).

17 Averroes (1967).

18 Aristotle (1933, pp. 139–145).

19 Kant (1943, pp. 331–359).

20 In fact, Leiter admits on p. 62 that philosophical reflection does not run the risk of commitment to a system of beliefs insulated from reasons and evidence.

21 Fox and others (1660).

22 Alberta *v*. Hutterian Brethren of Wilson Colony, 2009 SCC 37, [2009] 2 S.C.R. 567.

23 Of course, a certain strand of meta-ethical theory, known as ethical naturalism or naturalistic cognitivism, claims that moral properties are reducible to natural and non-moral properties. Leiter claims that, in this view, moral statements are answerable to evidence as understood in the natural sciences. We will discuss the implications of this claim for the alleged distinction between religious claims of conscience and secular claims of conscience in section “The Epistemology of Secular and Religious Conscientious Commitments”.

24 Moore (1903).

25 Miller (2003, p. 258); *Cf.* McDowell (1984). Leiter is aware of those alternative moral epistemologies. In a footnote, he dismisses, without arguing, McDowell's and all other non-naturalist types of moral realism as being implausible. He also recognizes that certain strands of Kantian ethics and of intuitionism ‘have quite a lot in common with religion’ (p. 153). However, he does not explain why this point does not jeopardize his argument for maintaining a sharp separation between religious prescriptions and secular moral obligations.

26 Railton (1986a, p. 16).

27 Railton (1986b, p. 190).

28 Dworkin (2013, pp. 22–23).

29 Ayer (1952, p. 115).

30 Nagel (2010, pp. 3–17).

31 Dworkin (2013, pp. 155–159).

32 Galeotti (1993, p. 587). See also Waldron (1988, p. 63), Williams (1996, p. 19), Scanlon (2003, p. 187).

33 Mendus (1988, p. 3).

34 For instance, Galeotti (2002, pp. 21–22).

35 Walzer (1997, p. 52).

36 Kymlicka (1992, pp. 34–35).

37 Locke (1998, p. 7).

38 Locke (1998, p. 39). For a similar interpretation of Locke, see Gutmann (2000, pp. 129–131), claiming that Locke, as well as Madison and Jefferson, understood freedom of religion to be ‘freedom within the limits of law that serve public purposes and that are not intended to limit religious freedom’ (at 131).

39 We follow Leiter in presenting toleration as a virtue of political institutions, even though many authors prefer to refer to the ideal of state neutrality.

40 Leiter also seems to understand toleration and freedom of conscience as freedom within the boundaries of generally applicable laws pursuing legitimate collective goals that do not directly aim at restricting freedom of conscience. He thus claims, for instance, that toleration allows the state to restrict freedom of conscience ‘as long as its objective in doing so is *not* to suppress or coercively burden those claims of conscience but to achieve some conception of the good’ (p. 117, *Cf.* 115, 124).

41 Bou-Habib (2006), Quong (2006), Seglow (2010), Shorten (2010), Boucher (2011), Maclure and Taylor (2011).

42 Sandel (2005, p. 257). Other theorists propose respect-based theories of religious freedom. See for instance, Modood (2010).

43 Rawls (1971, p. 207).

44 Mill (1999, Chapters 2 and 3).

45 Rawls (1971, p. 212).

46 Leiter could object that he examined principled arguments for toleration in order to solve the exemptions puzzle because toleration provides the moral foundation of religious liberty (see for instance p. 68). From this point of view, one must inquire into theories of toleration in order to determine whether exemptions are sometimes required to protect religious freedom and whether they should only be granted to accommodate religious claims. However, is it not more likely that religious liberty is the moral foundation of toleration (and not the other way around)? For instance, the Rawlsian argument asserts that the contracting parties of the original position would not choose an intolerant religious or laicist state because they prefer to protect their freedom of conscience.

47 Seglow (2010).

48 Quong (2006, p. 62).

49 Bou-Habib (2006), Maclure and Taylor (2011, pp. 76–77).

50 Eisgruber and Sager (2007, pp. 202–203), Nussbaum (2008, pp. 20, 116–119). .

51 Barry (2001, pp. 40–50). Barry’s view, however, is that exemptions are not required by justice but can nonetheless be required by prudence.

52 See Laborde (2008, p. 82).
